# Prevalent and Incident HIV Diagnoses among *Entamoeba histolytica*-Infected Adult Males: A Changing Epidemiology Associated with Sexual Transmission — Taiwan, 2006–2013

**DOI:** 10.1371/journal.pntd.0003222

**Published:** 2014-10-09

**Authors:** Yi-Chun Lo, Dar-Der Ji, Chien-Ching Hung

**Affiliations:** 1 Office of Preventive Medicine, Centers for Disease Control, Taipei, Taiwan; 2 Department of Internal Medicine, National Taiwan University Hospital and National Taiwan University College of Medicine, Taipei, Taiwan; 3 Center for Research, Diagnostics and Vaccine Development, Centers for Disease Control, Taipei, Taiwan; 4 Department of Tropical Medicine, National Yang-Ming University, Taipei, Taiwan; 5 Department of Medical Research, China Medical University Hospital, Taichung, Taiwan; 6 China Medical University, Taichung, Taiwan; Alfaisal University, Saudi Arabia

## Abstract

**Background:**

Sexually transmitted *Entamoeba histolytica* infection (EHI) has been increasingly recognized among men who have sex with men (MSM). We used the National Disease Surveillance Systems (NDSS) to identify prevalent and incident HIV diagnoses among adults with EHI and to determine the associated factors.

**Methodology:**

The NDSS collect demographic, clinical, and behavioral characteristics of case patients through physician reports and public health interviews. EHI was confirmed by polymerase-chain-reaction assays, histopathology, or serology with documented liver abscess. We linked NDSS databases to identify prevalent and incident HIV diagnoses among noninstitutionalized Taiwanese adults with confirmed EHI during 2006–2013. Cox proportional-hazards analysis was used to determine associated factors.

**Principal findings:**

Of noninstitutionalized adults with EHI, we identified prevalent HIV diagnosis in 210 (40%) of 524 males and one (1.7%) of 59 females, and incident HIV diagnosis in 71 (23%) of 314 males. MSM accounted for 183 (87%) and 64 (90%) of prevalent and incident HIV diagnoses in males, respectively. From 2006–2009 to 2010–2013, the prevalence of HIV diagnosis increased from 32% to 45% (*P* = 0.001) while the incidence of HIV diagnosis increased from 5.4 to 11.3 per 100 person-years (*P* = 0.001) among males with EHI. Incident HIV diagnosis was independently associated with a younger age, residing in metropolitan areas, hospitalization, previous syphilis, and engagement in oral, anal, or oral–anal sex before illness onset.

**Conclusions/significance:**

Prevalent and incident HIV diagnoses were increasingly identified among adult males in Taiwan, preferentially affecting younger urban MSM. Surveillance and risk-reduction interventions are recommended against the interplay of HIV epidemic and sexually transmitted EHI.

## Author Summary

Although sexually transmitted *Entamoeba histolytica* infection has been increasingly recognized among men who have sex with men (MSM) in Australia, Japan, Korea, and Taiwan, particularly those with HIV infection, no studies have examined *E. histolytica* infection in relation to HIV and attribution of sexual transmission at a national level. We analyzed the national surveillance data in Taiwan and demonstrated that the prevalent and incident HIV diagnoses increased among *E. histolytica*-infected adult males from 2006–2009 to 2010–2013, which were associated with previous syphilis and engagement of oral, anal, oral–anal sex. The findings indicate sexual transmission might have replaced traditional risk factors as the major route of *E. histolytica* transmission among adult males in Taiwan. *E. histolytica* infection associated with prevalent and incident HIV diagnoses preferentially affects younger urban MSM, suggesting new targets of surveillance and public health interventions for this sexually transmissible protozoan infection.

## Introduction

*Entamoeba histolytica* is a fecal-orally transmitted protozoan that generally causes asymptomatic infection but can lead to invasive diseases such as colitis and liver abscess [1], [2]. *E. histolytica* infection (EHI, also known as amebiasis) is endemic in areas of inadequate sanitation, particularly in the tropics. Traditionally, populations at increased risk for EHI include residents with lower socioeconomic status in endemic areas, immigrants or travelers from endemic areas, and institutionalized individuals with developmental or cognitive impairment [1]–[3]. Since the 1980s, sexually transmitted EHI has been increasingly reported among MSM, particularly those with human immunodeficiency virus (HIV) infection, from developed countries such as Australia, Japan, Korea, and Taiwan [3]–[12]. A case-control study and a cross-sectional study on voluntary counseling and testing (VCT) clients demonstrated *E. histolytica* seropositivity was associated with male–male sex, oral–anal contact, concomitant syphilis, and HIV coinfection [13], [14]. The results suggest that sexual risk behaviors predispose *E. histolytica*–infected individuals to acquisition of HIV infection and other sexually transmitted disease (STDs).

The increasingly recognized importance of sexual transmission of *E. histolytica* has been in line with recent studies that demonstrated high seroprevalence and incidence of EHI among MSM or HIV-infected individuals in East Asia [6], [11], [12], [15]–[18]. However, the prevalence and incidence of HIV infection among patients with EHI has not been adequately examined. In Taiwan, four hospital-based retrospective case series have demonstrated varied HIV prevalence (0%–85%) among patients with amebic liver abscess, but these studies were limited by the small case number and single-center design, and the wide range of HIV prevalence might be related to difference in the service population of the hospitals [19]–[22]. Furthermore, although both *E. histolytica* and HIV can be sexually transmitted, to our knowledge no study has assessed the incidence and determinants of HIV infection among *E. histolytica*-infected individuals. In this study, we aimed to use the national surveillance data to identify prevalent and incident HIV diagnoses among adults with EHI and to determine the factors associated with HIV diagnosis.

## Methods

### Surveillance and investigations of EHI

The Communicable Disease Control Act in Taiwan has mandated physicians to notify local public health departments of suspected or confirmed cases of EHI within 24 hours of diagnoses since 1983. Physicians are required to report demographic and clinical characteristics through the web-based, Taiwan Centers for Disease Control (TCDC)-operated Notifiable Disease Surveillance Systems (NDSS). In all reported cases, TCDC require three fresh stool samples, each collected at ≥24-hour intervals within seven days after reporting and stored at 4°C, and abscess aspirates (if available) to be submitted within 24 hours of specimen collection to the TCDC laboratory for *E. histolytica*-specific polymerase-chain-reaction (PCR) assay that has replaced microscopic examination as the confirmatory method of EHI since 2004. Methods of *E. histolytica*-specific PCR assay have been described elsewhere [11], [23].

TCDC defines a suspected EHI case as presence of any of the following conditions: (1) mucous or bloody diarrhea with or without abdominal pain, fever, nausea, or vomiting; (2) microscopic detection of cysts or trophozoites suspicious of *E. histolytica*; and (3) clinical and radiological evidence of amebic liver abscess. A confirmed EHI case is defined as presence of any of the following conditions: (1) positive *E. histolytica*-specific PCR assay in clinical specimens; (2) histopathologic diagnosis of invasive amebiasis based on detection of *E. histolytica* trophozoites in tissues; and (3) clinical and radiological evidence of amebic liver abscess with positive *E. histolytica* serology based on indirect hemagglutination antibody (IHA) testing (Cellognostics, Boehringer Diagnostics GmbH, Marburg, Germany) at clinical laboratories.

For all confirmed EHI cases, local health department staff initiated immediate case investigations through face-to-face or telephone interviews. A standardized questionnaire was administered to collect self-reported information including residing institutions, countries of origin, recent travel to endemic areas, and clinical presentations. Patients were also asked if he or she had engaged in oral, anal, or oral–anal sex before illness onset. The responses were entered into the NDSS database.

### Surveillance and investigations of HIV infection

Since 1985, TCDC has mandated medical professionals to notify local public health departments of cases of HIV infection and acquired immunodeficiency syndrome (AIDS) within 24 hours of diagnoses. Demographic and clinical characteristics are reported through the NDSS. HIV infection was defined as positive HIV-1 Western blot or PCR testing conducted at TCDC or TCDC-certified laboratories at designated hospitals for HIV care. AIDS was defined as presence of a CD4 lymphocyte count <200 cells/mm^3^ or any of the AIDS-defining conditions in an individual with HIV infection [24].

For all reported cases, TCDC requires local health department staff to initiate case investigations through face-to-face interviews within seven days of reporting. A standardized questionnaire is administered to collect self-reported information on sexual behaviors, injection drug use, and other risk factors such as transfusion and mother-to-child transmission. Since April 1997, TCDC has offered free-of-charge highly active antiretroviral therapy (HAART) and HIV-related clinical, laboratory, prevention and treatment services through designated hospitals to all reported HIV-infected Taiwanese nationals [25]. Local health department professionals and/or specialized case managers at designated hospitals are required to follow up reported HIV patients every three to six months, collect information on the latest CD4 count, plasma HIV RNA load (PVL), and use of HAART from the patients or health-care providers, and enter the data into the NDSS-HIV database.

### Surveillance of syphilis and gonorrhea

In Taiwan, syphilis and gonorrhea have been included in the nationally notifiable diseases since 1999. Physicians are required to report laboratory-confirmed cases of syphilis and gonorrhea through the NDSS within seven days of diagnoses. TCDC defines laboratory-confirmed syphilis as presence of any of the following conditions: (1) a reactive nontreponemal test with a *Treponema pallidum* hemagglutination or particle agglutination assay titer ≥1∶320; and (2) fourfold or greater increase of a nontreponemal test titer in a person previously treated for syphilis. Laboratory-confirmed gonorrhea is defined as identification of *Neisseria gonorrhoeae* in clinical specimens by culture or PCR assays, or observation of gram-negative diplococci in genitourinary specimens by microscopy.

### Data collection and database linkage

We collected demographic, clinical, and behavioral characteristics of patients with confirmed EHI reported during 2006–2013 from the NDSS. We linked the NDSS databases of EHI, HIV infection, syphilis, and gonorrhea using a government-issued, non-duplicated national identification number that is unique and compulsory for each Taiwanese national. The 1999–2013 NDSS databases of syphilis and gonorrhea were linked to identify reports of syphilis or gonorrhea before the diagnosis of EHI. The 1985–2013 NDSS-HIV databases were linked to identify reports of HIV infection among the confirmed EHI cases and collect demographic, clinical, and laboratory characteristics of patients with HIV infection.

### Analysis criteria

The analysis used a retrospective cohort design with the assumption that *E. histolytica*-infected Taiwanese nationals were a closed population. To avoid duplicated representation of the same individuals, we used the national identification number to identify individuals with two or more reports of confirmed EHI in the NDSS, and included only the earliest report during 2006–2013 for each of these individuals. For this analysis, non-Taiwanese nationals were excluded because they did not have a national identification number for database linkage.

The linkage of NDSS databases were conducted in all Taiwanese nationals aged ≥18 years with confirmed EHI. We defined prevalent HIV diagnosis as an HIV diagnosis before or on the same date of an EHI diagnosis, and incident HIV diagnosis as an HIV diagnosis after the date of an EHI diagnosis. Individuals were defined as residing in metropolitan areas if they reported residing in any of the six major municipalities (Taipei, New Taipei, Taoyuan, Taichung, Tainan, and Kaohsiung) at the time of *E. histolytica* diagnosis. *E. histolytica*-endemic areas were defined as Asia (excluding Japan, Republic of Korea, Taiwan, Hong Kong, and Macau), Africa, Mexico, Central and South America, and the Pacific Islands [3]. Data of the National Death Registry based on death certificates issued by healthcare providers were linked to identify deaths during the follow-up period.

### Statistical analysis

We defined the incidence rate as the number of cases of incident HIV diagnosis divided by the total person-years of follow-up (PYFU). The follow-up period was estimated as the period from the first EHI diagnosis after January 1, 2006 to the date of HIV diagnosis, death, or December 31, 2013, whichever occurred first. We conducted bivariate analysis to compare characteristics of patients with EHI using the chi-square test or Fisher's exact test for categorical variables. The trend in prevalence was evaluated with the chi-square test for trend. A univariate Cox proportional-hazards analysis was used to estimate the impact of baseline characteristics on the incidence of HIV diagnosis. Variables that were associated with incident HIV diagnosis (p<0.05) in univariate analyses were considered candidates in a multivariable Cox proportional-hazards model. We used adjusted hazard ratio (aHR) and 95% confidence intervals (CIs) to estimate the impact of individual characteristics on incident HIV diagnosis. The analyses were conducted with the statistical software package SAS version 9.2 (SAS Institute, Inc., Cary, North Carolina).

### Ethics statement

Data obtained for the NDSS was for public health surveillance purposes. This study was approved by the TCDC Institutional Review Board. The patient data used in this study was anonymized.

## Results

Of 4,517 reported EHI cases to TCDC through the NDSS during 2006–2013, 1,742 (38.6%) were confirmed cases which occurred in 1,723 individuals, including 870 (50.5%) non-Taiwanese and 853 (49.5%) Taiwanese nationals. Of 870 non-Taiwanese, the most prevalent nationalities were Indonesia (81.8%), the Philippines (9.4%), Vietnam (4.6%) and Thailand (1.5%), and 820 (94.3%) had reported recent immigration or travel from endemic areas. Of 853 Taiwanese nationals, 848 were aged ≥18 years and were included in the analysis.

### Trend of prevalent HIV diagnosis among adults with EHI

**Figure 1:**
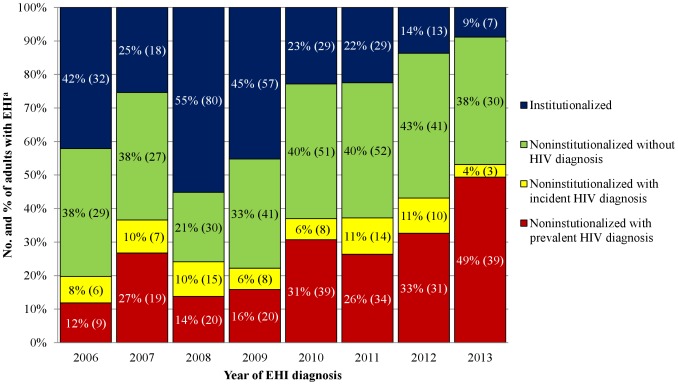
Trend of adults with confirmed *Entamoeba histolytica* infection (EHI) by population at risk, Taiwan, 2006–2013. ^a^Numbers in parentheses indicate numbers of EHI cases in each risk category.

Of 848 Taiwanese adults with EHI, 265 (31.2%) were institutionalized and 583 (68.8%) were noninstitutionalized. Prevalent HIV diagnosis was identified in 211 (24.9%) adults, all of whom were noninstutionalized. The prevalence of HIV diagnosis among *E. histolytica*-infected adults increased from 12% in 2006 to 49% in 2013 (p<0.001), whereas the proportion of institutionalized adults decreased from 42.1% in 2006 to 8.9% in 2013 (p<0.001) (Figure 1). Adults with prevalent HIV diagnosis had predominated over the institutionalized adults in EHI cases since 2010.

**Figure 2:**
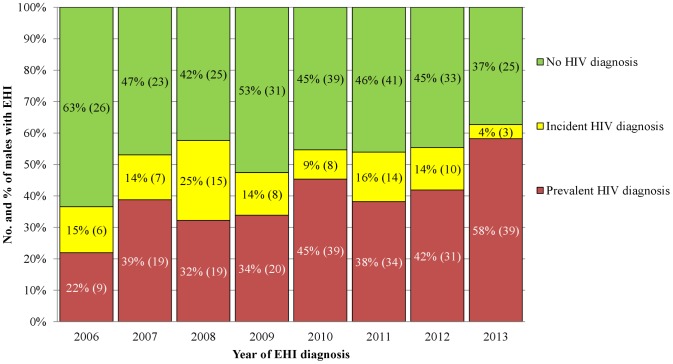
Trend of noninstitionalized adult males with confirmed *Entamoeba histolytica* infection by HIV diagnosis, Taiwan, 2006–2013. ^a^Numbers in parentheses indicate numbers of EHI cases in each risk category.

In a separate analysis that included only noninstitutionalized Taiwanese adults with EHI, prevalent HIV diagnosis was identified in 210 (40%) of 524 males and only one (1.7%) of 59 females. Among the noninstitutionalized adult males, the prevalence of HIV diagnosis increased from 22% in 2006 to 58% in 2013, and the overall prevalence of HIV diagnosis during the periods of 2006–2009 and 2010–2013 increased from 32% to 45% (p = 0.001) (Figure 2).

### Characteristics of males with prevalent HIV diagnosis

Of 210 males with prevalent HIV diagnosis, the median age was 35 (range, 20–79) years at the time of EHI diagnosis. Prevalent HIV diagnosis was made within a median of 908 (range, 0–7,744) days before EHI diagnosis. The majority of males with prevalent HIV diagnosis were aged 18–40 years (70%), residents in metropolitan areas (86%), and hospitalized (59%) at the time of EHI diagnosis; only 11% reported having traveled to endemic areas before the illness onset (Table 1). Approximately half (52%) had been reported with syphilis and 40% had engaged in oral, anal or oral–anal sex before illness onset. MSM accounted for 183 (87%) males with prevalent HIV diagnosis.

**Table 1:** Characteristics of *Entamoeba histolytica*-infected noninstitutionalized Taiwanese adult males in relation to HIV diagnosis — Taiwan, 2006–2013.

Characteristics at EHI diagnosis	(A) Prevalent HIV diagnosis (N = 210) No. (%)	(B) Incident HIV diagnosis (N = 71) No. (%)	(C) No HIV diagnosis (N = 243) No. (%)	*P* Value (A) vs. (C)	*P* Value (B) vs. (C)
Age group (year)					
18–30	53 (25)	26 (37)	34 (14)	<0.001	<0.001
31–40	95 (46)	30 (42)	64 (26)		
41–50	52 (25)	11 (15)	53 (22)		
>50	10 (5)	4 (6)	92 (38)		
Residing in metropolitan areas	180 (86)	62 (87)	175 (72)	<0.001	0.008
Recent travel to endemic areas	24 (11)	12 (17)	45 (19)	0.04	0.76
Hospitalized	124 (59)	48 (68)	124 (51)	0.09	0.01
Amebic liver abscess	89 (42)	31 (44)	93 (38)	0.37	0.41
Previous syphilis report	109 (52)	17 (24)	15 (6)	<0.001	<0.001
Previous gonorrhea report	8 (4)	6 (8)	3 (1)	0.14	0.01
Oral, anal or oral-anal sex before illness onset	83 (40)	20 (28)	22 (9)	<0.001	<0.001

Abbreviations: EHI, *Entamoeba histolytica* infection.

Of 196 (93%) males with prevalent HIV diagnosis and available laboratory data within three months of EHI diagnosis in the NDSS, the median CD4 count was 327 (range, 1–1421; 60 [31%] with CD4 count <200) cells/mm^3^ and the median PVL was 2.65×10^4^ (range, <20 to 9.88×10^6^; 31 [16%] with an undetectable PVL) copies/ml at the time of EHI diagnosis, and 66 (34%) had received HAART before EHI diagnosis.

In bivariate analysis that compared characteristics of adult males in relation to HIV diagnosis, prevalent HIV diagnosis was associated with a younger age (median, 35 vs. 44 years, p<0.001), residing in metropolitan areas, no recent travel to endemic areas, previously syphilis reports, and engagement of oral, anal or oral–anal sex before illness onset (Table 1).

### Incident HIV diagnosis

Of the 372 noninstitutionalized Taiwanese adults without prevalent HIV diagnosis, incident HIV diagnosis occurred in 71 (23%) of 314 males aged 18–69 years (median, 33 years) within a median of 10 (range, 1–1,961) days after EHI diagnosis, and in none of 58 females. MSM accounted for 64 (90%) of 71 males. During the follow-up period, 13 deaths occurred 3–141 days after EHI diagnosis. The total duration of follow-up was 1,161 PYFU (mean 3.1 PYFU). We estimated that the overall incidence rate of HIV diagnosis after EHI was 6.1 per 100 PYFU (95% CI, 4.8–7.7) and the incidence rate in males was 7.3 per 100 PYFU (95% CI, 5.7–9.1). From the period of 2006–2009 to 2010–2013, the incidence rate of HIV diagnosis after EHI in males increased by 2.1 fold, from 5.4 per 100 PYFU (95% CI, 3.8–7.4) to 11.3 per 100 PYFU (95% CI, 8.0–15.6) (p = 0.001).

Of 62 (87%) males with available laboratory data within three months of incident HIV diagnosis in the NDSS, the median CD4 count was 223 (range, 2–892; 29 [47%] with CD4 count <200) cells/mm^3^ and the median PVL was 5.34×10^4^ copies/ml (range, 1.66×10^2^–9.55×10^5^ copies/ml) at the time of incident HIV diagnosis. AIDS was concomitantly diagnosed in 31 (50%) of the patients.

To avoid confounding of sex, only males were included in the Cox proportional-hazards analysis. In the multivariable model, *E. histolytica*-infected males were more likely to have incident HIV diagnosis if they were aged 18–30 years (aHR, 12.6; 95% CI, 4.3–36.8), 31–40 years (aHR, 10.2; 95% CI, 3.5–29.6), or 41–50 years (aHR, 3.6; 95% CI, 1.1–11.3); resided in metropolitan areas (aHR, 2.8; 95% CI, 1.3–5.8); were hospitalized (aHR, 6.5; 95% CI, 3.6–11.9), had been reported with syphilis (aHR, 3.9; 95% CI, 2.0–7.4), and had engaged in oral, anal, or oral–anal sex before illness onset (aHR, 2.7; 95% CI, 1.5–4.9) (Table 2). To examine whether the risk estimates might be affected by the definition used or delayed reporting, we performed a sensitivity analysis using three alternative definitions of incidence HIV diagnosis: ≥7 days, ≥14 days, and ≥28 days after EHI diagnosis. Risk estimates, except for the variable of hospitalization, were generally similar to the results from the main analysis, with wider confidence intervals due to smaller number of cases (Table S1).

**Table 2:** Risk analysis for incident HIV diagnosis among *Entamoeba histolytica*-infected noninstitutionalized Taiwanese adult males by Cox proportional-hazards model.

Characteristics at EHI Diagnosis	Unadjusted HR (95% CI)	Adjusted HR (95% CI)
Age group (years)		
18–30	12.9 (4.5–37.1)	12.6 (4.3–36.8)
31–40	8.6 (3.0–24.5)	10.2 (3.5–29.6)
41–50	4.3 (1.4–13.6)	3.6 (1.1–11.3)
>50	reference	reference
Residing in metropolitan areas		
Yes	2.4 (1.2–4.8)	2.8 (1.3–5.8)
No	reference	reference
Recent travel to endemic areas		
Yes	0.9 (0.5–1.6)	
No	reference	
Hospitalized		
Yes	2.0 (1.2–3.2)	6.5 (3.6–11.9)
No	reference	reference
Amebic liver abscess		
Yes	1.3 (0.8–2.1)	
No	reference	
Previous syphilis report		
Yes	3.4 (2.0–5.9)	3.9 (2.0–7.4)
No	reference	reference
Previous gonorrhea report		
Yes	4.0 (1.7–9.4)	1.2 (0.5–2.9)
No	reference	reference
Oral, anal or oral-anal sex before illness onset		
Yes	3.0 (1.8–5.0)	2.7 (1.5–4.9)
No	reference	reference

Abbreviations: EHI, *Entamoeba histolytica* infection; HR, hazards ratio.

## Discussion

This study based on NDSS data demonstrates trends of increasing prevalent and incident HIV diagnoses among *E. histolytica*-infected noninstitutionalized Taiwanese adult males. Prevalent or incident HIV diagnoses occurred predominantly in MSM aged 18–40 years who resided in metropolitan areas, and were associated with prior syphilis and practices of oral, anal or oral–anal sex. These findings suggest sexual transmission, principally male–male sex, contributed substantially to the case burden of amebiasis and might have become a major route of *E. histolytica* transmission in Taiwan. Over the 8-year study period, a changing epidemiology of EHI was demonstrated. Although the estimated number of institutionalized individuals in Taiwan increased from 65,670 in 2006 to 73,994 in 2011 [26], [27], the number of patients with EHI who were institutionalized during our study period has significantly decreased, likely attributed to continued efforts of eradication of *E. histolytica* in large psychiatric institutions by annual or semi-annual intensive surveillance with mass screening and treatment [28]. In contrast, the number of patients with EHI who were HIV-infected has increased by approximately twofold and HIV-infected individuals have replaced institutionalized individuals as the major risk groups. The increased burden of prevalent HIV diagnosis among EHI cases occurred simultaneously with the rapidly emerging HIV epidemic in Taiwan that has preferentially affected MSM [27]. The annual number of newly reported HIV cases increased by 28%, from 1,752 (including 999 [57%] MSM) in 2008 to 2,244 (including 1,821 [81%] MSM) in 2013 [29]–[31]. The estimated HIV prevalence among adults aged 15–49 years increased by 31%, from 0.16% in 2008 to 0.21% in 2012 [32], [33]. Furthermore, since 2008, the national HIV treatment guidelines have recommended routine *E. histolytica* IHA testing at the diagnosis of HIV infection, particularly for HIV-infected MSM [34]. This recommendation might have also contributed to increased detection of EHI associated with prevalent HIV diagnosis.

The prevalence (12–49%) and incidence (5.4–11.3 per 100 PYFU) of HIV diagnoses among males with EHI in our study were higher than recent estimates of HIV prevalence (8.1%–10.7%) and incidence (4.6–6.3 per 100 PYFU) among MSM [35], [36]. Because using prevalent HIV diagnosis as a surrogate marker would underestimate the true HIV prevalence, our findings indicate that *E. histolytica*-infected males carry a higher risk for prevalent HIV infection than MSM. However, cautions are needed in the comparison of incidence rates. Because temporal relationships between HIV acquisition and EHI were not determined, incident HIV diagnosis might have underrepresented incident infection if HIV infection after EHI was undiagnosed, and might have misrepresented prevalent infection if EHI occurred between HIV infection and HIV diagnosis. Alternative definitions of incident HIV diagnosis would also affect the estimated incidence of HIV diagnosis and risk estimates, but our sensitivity analysis demonstrated similar risk estimates for all variables except for the hospitalization variable. A prospective cohort study with HIV testing at baseline and regular intervals would determine true HIV incidence among EHI cases for comparison with other populations at risk.

Although sexually transmitted EHI is preventable by avoiding direct and indirect oral–anal contact, recommendations of preventive measures (e.g. use of condoms, gloves, and dental dams during sex, including use of gloves for “fisting”) have been solely based on expert opinions and require directly applicable studies [37]. Awareness of *E. histolytica* disease and transmission has not been assessed among MSM and HIV-infected individuals. Barriers to adoption of safe or safer sex practices, such as challenges in communicating with partners and stigmatization associated with condom use [38], likely exist in the scenario of oral–anal contact as well and should be identified to facilitate development of effective risk-reduction interventions against *E. histolytica* transmission. Although *E. histolytica* has not been shown to transmit HIV or increased host susceptibility to HIV infection, HIV-related immunosuppression might increase susceptibility of HIV-infected individuals to invasive *E. histolytica* disease such as amebic liver abscess [39], [40]. Continuous public health surveillance efforts are warranted in preparation for possible EHI outbreaks accompanied by the ongoing HIV epidemic. Health-care providers should include EHI-related risk-reduction counseling in the integrated care and services for newly diagnosed HIV-infected individuals. Sexually active HIV-infected individuals should be counseled to avoid sexual practices that may facilitate fecal-orally transmitted infections including EHI.

Almost half (47%) of the incident HIV diagnosis met the definition of late HIV diagnosis (CD4<200 cells/mm^3^), indicating possible missed opportunities for early HIV detection in these patients [41]. Their clinical and public health encounters for EHI might have served as a valuable opportunity for HIV testing and counseling. We suggest that health-care providers take sexual history for all *E. histolytica*-infected adult males without traditional risk factors, assess STD/HIV risk, and offer HIV testing and counseling at clinics if sexual risk is identified. Besides promotion of hand hygiene and sanitation, public health professionals are recommended to conduct partner notification, provide risk-reduction counseling about EHI and STD/HIV, and introduce STD/HIV prevention services among *E. histolytica*-infected males without traditional risk factors. Based on our study findings, HIV testing may be prioritized to *E. histolytica*-infected males aged 18–40 years in metropolitan areas and be repeated every 3–6 months for early HIV detection if ongoing sexual risk is present. Educational activities are recommended towards health-care providers (e.g., gastroenterologists, internists, family physicians) and public health professionals serving patients with EHI to establish or strengthen their knowledge, skills, and confidence in STD/HIV assessment and counseling.

In this study, MSM status and all behavioral data were self-reported, collected through face-to-face or telephone interviews, and thus subject to recall, interviewer, and socially-desirable biases. The public health investigators were not specifically trained to avoid unintentional influence on respondents' answers to sensitive questions, whereas respondents might have been reluctant to disclose sensitive information to interviewers. These biases might have led to underreported male–male and oral, anal, or oral–anal sex. Audio computer-assisted self-interviews, a self-report method commonly used to assess sexual behaviors worldwide but rarely used in Taiwan, might enhance privacy, reduce socially desirable responses, and improve accuracy of the collected behavioral data [42]–[46].

Interpretation of the study results should take the following limitations into account. First, because confirmed EHI cases require laboratory or pathologically diagnostic evidence, the surveillance data is expected to underestimate *E. histolytica* disease burden and overrepresent amebic liver abscess which accounted for 43% of our NDSS sample. Similarly, data on previous syphilis and gonorrhea reports are likely underestimated because diagnoses could be made clinically without laboratory confirmation, leading to underreporting. Second, the association between EHI hospitalization and incident HIV diagnosis was likely confounded by coexisting undiagnosed HIV-related complications or provision of HIV testing as part of the investigation of hospitalized EHI cases. The reduced risk estimate for the hospitalization variable in our sensitivity analysis supports the hypothesis of presence of unexamined confounding events that occurred during EHI hospitalization. Furthermore, because patients were inquired about engagement of oral, anal, and oral–anal sex practices as one question and not about male–male sex, we could not determine the attributable impact of oral–anal sex or male–male sex on EHI. Finally, a substantial proportion of EHI cases occurred in noninstitutionalized adult males without HIV diagnosis who were characterized by an older median age and significantly lower proportions of residing in metropolitan areas, previous syphilis reports, and oral, anal, or oral–anal sex than males with HIV diagnosis. However, except for males who reported recent travel to endemic areas or engagement in oral, anal, or oral–anal sex, the sources of EHI were not determined. Whether these cases are attributed to sexual, foodborne, or waterborne transmission warrants further investigations.

In conclusion, the epidemiology of EHI in Taiwan has changed during 2006–2013 with preponderance of younger urban MSM infected with or at increased risk for HIV infection as the major affected population. Surveillance and risk-reduction interventions are recommended against the interplay of HIV epidemic and sexually transmitted EHI in Taiwan.

## Supporting Information

Checklist S1 STROBE checklist.
(DOCX)

Table S1 Risk estimates by Cox proportional-hazards model in the main analysis and the sensitivity analysis using three alternative definitions of incidence HIV diagnosis: ≥7 days (a), ≥14 days (b), and ≥28 days (c) after EHI diagnosis among noninstitutionalized Taiwanese adult males.
(DOCX)
