# Supplementary material for: Prevalent and Incident HIV Diagnoses among Entamoeba histolytica-Infected Adult Males: A Changing Epidemiology Associated with Sexual Transmission — Taiwan, 2006–2013
Source: PLoS Negl Trop Dis. 2014 Oct 9;8(10):e3222. doi: 10.1371/journal.pntd.0003222 (PMC4191956; doi:10.1371/journal.pntd.0003222)
Supplement: Table S1 — Risk estimates by Cox proportional-hazards model in the main analysis and the sensitivity analysis using three alternative definitions of incidence HIV diagnosis: ≥7 days (a), ≥14 days (b), and ≥28 days (c) after EHI diagnosis among noninstitutionalized Taiwanese adult males. (DOCX) [file pntd.0003222.s002.docx]

Supplementary Table. Risk estimates by Cox proportional-hazards model in the main analysis and the sensitivity analysis using three alternative definitions of incidence HIV diagnosis: ≥7 days (a), ≥14 days (b), and ≥28 days (c) after EHI diagnosis among noninstitutionalized Taiwanese adult males

| Characteristics at EHI diagnosis | Incident case defined as HIV diagnosis ≥x day(s) after EHI diagnosis | | | |
| --- | --- | --- | --- | --- |
|  | Main analysis, x=1  aHR (95% CI) | Sensitivity (a), x=7  aHR (95% CI) | Sensitivity (b), x=14  aHR (95% CI) | Sensitivity (c), x=28  aHR (95% CI) |
| Age group (years) |  |  |  |  |
| 18–30 | 12.6 (4.3–36.8) | 13.7 (4.0–47.1) | 16.4 (3.7–73.1) | 24.0 (3.1–188) |
| 31–40 | 10.2 (3.5–29.6) | 8.7 (2.5–30.3) | 7.0 (1.5–32.7) | 11.0 (1.4–89.1) |
| 41–50 | 3.6 (1.1–11.3) | 3.5 (0.9–13.7) | 1.5 (0.2–10.7) | 1.4 (0.1–22.2) |
| >50 | reference | reference | reference | reference |
| Residing in metropolitan areas |  |  |  |  |
| Yes | 2.8 (1.3–5.8) | 2.5 (1.1–5.9) | 2.2 (0.8–5.9) | 2.0 (0.6–6.2) |
| No | reference | reference | reference | reference |
| Hospitalized |  |  |  |  |
| Yes | 6.5 (3.6–11.9) | 4.0 (2.0–8.2) | 1.9 (0.9–4.3) | 1.5 (0.6–3.8) |
| No | reference | reference | reference | reference |
| Previous syphilis report |  |  |  |  |
| Yes | 3.9 (2.0–7.4) | 3.6 (1.6–8.0) | 2.4 (0.9–6.2) | 2.7 (1.04–7.2) |
| No | reference | reference | reference | reference |
| Oral, anal or oral-anal sex before illness onset |  |  |  |  |
| Yes | 2.7 (1.5–4.9) | 2.1 (0.9–4.4) | 1.6 (0.7–4.1) | 2.0 (0.6–6.2) |
| No | reference | reference | reference | reference |

Abbreviations: EHI, *Entamoeba histolytica* infection; aHR, adjusted hazards ratio
